# Development and Validation of the Scale for Partnership in Care—for Family (SPIC-F)

**DOI:** 10.3390/ijerph17061882

**Published:** 2020-03-13

**Authors:** Hye-Young Jang, Eun-Ok Song

**Affiliations:** College of Nursing, Hanyang University, Seoul 04763, Korea; wkqjr100@hanyang.ac.kr

**Keywords:** instrument development, partnership practice, family caregivers, nursing homes, older adults

## Abstract

This study aims to develop and validate the Scale for Partnership in Care between staff and families of older adult nursing home (NH) residents—for Family (SPIC-F). The components of partnership were identified on the basis of literature reviews and focus group interviews. The content validity of 41 preliminary items was verified by 10 experts, and a pilot study was conducted. The reliability and validity of the instrument was tested on 330 families of older adult NH residents. The final instrument comprised 20 items in three categories: professional caring and support, cooperative relationship and information sharing, and participation in care. Each item is rated on a four-point Likert scale, with total scores ranging from 20–80. The reliability of the instrument was 0.95, and test–retest ICC was 0.83. This instrument could be utilized to develop interventions to establish an efficient partnership and assess its outcomes.

## 1. Introduction

Population aging is a global phenomenon. As of 2019, the world’s population aged 65 or over was 703 million people and is expected to double to 1.5 billion by 2050 [1]. In particular, population aging in Northeast Asia is a notable phenomenon. In 2015, the number of elderly people aged 65 or older living in six Northeast Asian countries accounted for 32% of the world’s elderly population and accounted for 56% of Asia [2]. This aging phenomenon has led to an increase in geriatric diseases such as dementia, stroke, and cardio-cerebrovascular diseases, which ultimately lead to an increase in the population in need of care. Globally, the high prevalence of older people and the statistics of increased chronic disease, such as dementia, support these changes [3,4]. The increasing number of older adults in need of continuous protection and care raises various social and economic problems, including the burden on family and the increase in medical care costs [5,6].

Particularly, because policies prioritize home care, older adults can live and receive care in a familiar environment, which can lead family caregivers to experience serious difficulties [5,7]. These difficulties may include physical problems such as chronic headaches and fatigue [7]; mental problems such as stress, depression, and anxiety [5,8]; family conflicts due to care [5]; and the economic burden of care [7].

With such a heavy caregiving burden, families reach their limit in caring for older adults at home [9]. As a result, despite the negative perception of institutionalizing [5], the admission of older adults to nursing homes (NHs) has consistently risen, reaching 345,000 users of NHs in Korea in 2016 [6].

The common belief is that families’ caregiving burdens would be diminished by admitting their relatives to NHs, however, families of older adult NH residents still experience different aspects of the caregiving burden [10], such as guilt, confusion with their caregiving role, and role conflicts with NH staff [9,11,12].

Following older adults’ institutionalization, families delegate their role as primary caregiver to the NH staff and wish to continue to be involved in the care as the older adults’ advocate and watcher [13]. However, learning and adjusting to their changed role in a new environment is a difficult process [14]. Furthermore, there is no clear-cut definition of families’ new roles, which might isolate them or place them in a vague position in the caregiving service system [15,16,17].

Families of older adult residents may provide important information about residents’ habits, preferences, and care needs, resulting in the provision of high-quality care to older adult residents [18,19]. Therefore, the family’s participation in care is crucial for the wellbeing of the older adult residents [14], and it is important to come up with plans to help families become involved in care as partners rather than passive watchers.

Previous research on partnership in nursing includes studies that explored the meaning and analyzed the construction of partnership [20,21,22,23], studies that developed a partnership model [24,25,26,27], and studies that examined parental and family involvement [28,29]. Building an efficient partnership between families and staff helps to manage care for older adult residents that enhances their quality of life [18,30].

Despite the awareness of the importance of partnership, efforts to develop a standardized instrument for partnership assessment have been lacking. Tools developed thus far only assess some concepts related to partnership, such as trust [31] or treatment alliance [32], with no instrument encompassing the major components of partnership. Kiriake and Moriyama [33] developed a partnership assessment tool for families of patients with dementia that used a community daycare center. However, it focused on the families of patients with dementia; thus, there are limitations to using this instrument with families of older adults without dementia. Consequently, this tool cannot directly assess the effect of nursing interventions to promote partnership between the staff and families of older adult residents.

Therefore, this study aims to develop a tool for measuring partnership between the families of older adult residents and staff in the nursing home on the basis of the Wiggins’ Partnership Care Delivery Model [PCDM] [26,27]. According to Wiggins [26,27], the PCDM is a system of care that has safe patient- and family-centered care at its core, with all the disciplines engaged in a partnership to provide patient-centered care. The components of the PCDM include education and support, collaborative practice, and effective communication. In addition, successful collaboration consists of communication and interpersonal relationships based on trust and time.

Thus, the purpose of this study was to develop and validate an instrument to be administered to families of NH residents to assess their partnership with the NH staff on the basis of PCDM. This will not only identify the degree of partnership between the family member and staff, but also be useful for developing an intervention program for partnership formation in practice. In addition, it can be used as an indicator for quality management of facility care in terms of policy and can be used as basic data for evaluating and preparing improvement plans.

## 2. Methods

### 2.1. Study Participants

The participants were family members of older adult NH residents. The inclusion criteria were as follows: (1) families who most frequently visited the NHs after the older adults’ admission, (2) those who provided informed consent to participate in the study. Based on an appropriate sample size of 150–200 for exploratory factor analysis (EFA) [34], and at least 150 for confirmatory factor analysis (CFA) [35], we set the sample size to 300. Considering a 20% dropout rate, we collected data from 360 participants, and after excluding 30 questionnaires with inappropriate responses or withdrawal, a total of 330 questionnaires were analyzed.

### 2.2. Development of Instrument

**Figure 1:**
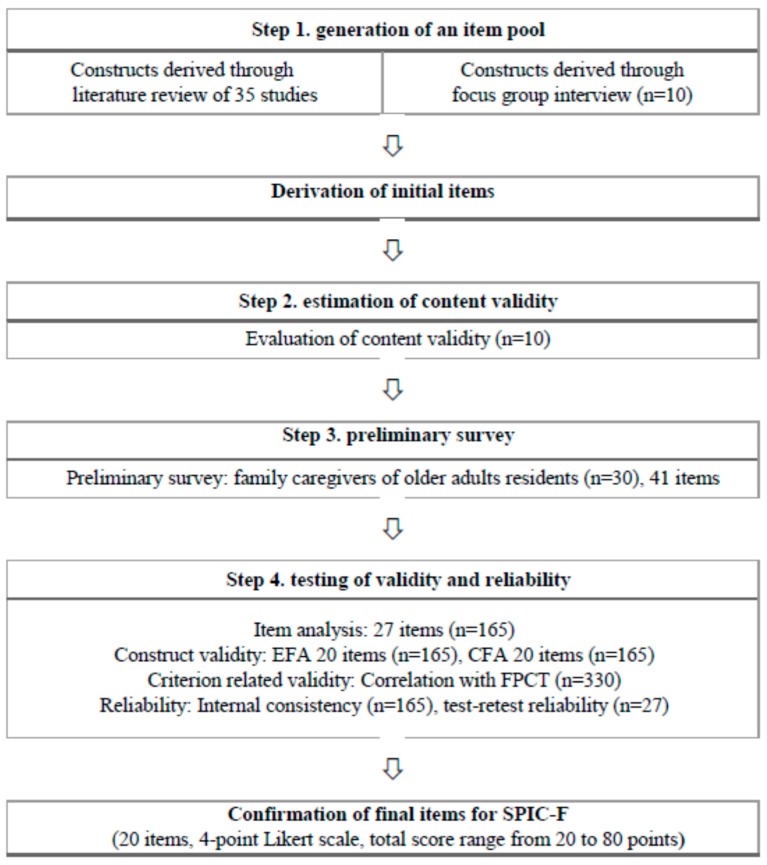
The SIPC-F development process. Notes. EFA = exploratory factor analysis; CFA = confirmatory factor analysis; FPCT = family perceptions of care tool; SPIC-F = scale for partnership in care—for family.

The Scale for Partnership in Care –for Family (SPIC-F) was developed in four stages based on the guidelines of DeVellis [36] on tool development (Figure 1).

#### 2.2.1. Item Generation

The item generation used a combination of deductive and inductive methods [37]. We used a literature review as the deductive method [38] and conducted a focus group interview (FGI) as the inductive method [39].

To review the literature pertaining to the concept of partnership, two researchers performed searches independently. PubMed, Embase, Cumulative Index to Nursing and Allied Health Literature (CINAHL), PsycInfo, Cochrane Library, Dissertation Abstracts, Research Information Sharing Service (RISS), and Korean studies Information Service System (KISS) were searched using the search terms “family”, “staff”, “partnership”, “nursing homes”, and “long-term care facility” to identify articles published between January 1980 and March 2017. The language was set to Korean and English. A total of 35 articles dealing with the construct of partnership were analyzed.

FGIs were conducted with 10 family members of older adult NH residents on September 2 and 30, 2017 to reconfirm the components of partnership identified in the literature review and identify additional components. To ensure an effective interaction between the participants [40,41], each group comprised five participants. The FGIs were conducted in a quiet conference room in the NH and lasted about 90 min on average. Data saturation was reached when no new information was discovered. Individual in-depth interviews were conducted with two FGI participants to complement and verify the results of the FGIs. The collected data were analyzed via qualitative content analysis [42]. Meaningful words, phrases, and sentences were coded by repeatedly reading the interview transcriptions. The differences and similarities among the codes were compared to extract categories that clustered the data in terms of relevance. Considering the connection and relevance among the categories, broader topics that were abstract and significant were extracted as the components of partnership.

The partnership components identified through the literature review and FGIs were relationship, sharing information, sharing decision-making, professional competence, and involvement in care. We developed 32 preliminary self-report items based on the identified components. Each item was rated on a 4-point Likert scale (1 = strongly disagree, 2 = disagree, 3 = agree, and 4 = strongly agree). To prevent fixed responses, items for the same construct were arranged nonconsecutively, and reverse-coded items were included. A higher score indicated a higher level of partnership between staff and families of older adult NH residents.

#### 2.2.2. Content Validity

Content validity was tested by a panel of 10 experts to determine the degree to which each item fit the operational definition of the construct. A panel of experts was formed with five nursing professors in the field of gerontological nursing with five or more years of experience and three nursing home managers and two nurses with 10 or more years of experience in the provision of care at a nursing home. These experts possessed a wealth of knowledge on family caregivers and nursing home residents. The content validity index (CVI) of the preliminary items was rated on a 4-point scale, and ratings of 3 (relevant) and 4 (very relevant) were processed as 1, and ratings of 2 (not relevant) and 1 (not relevant at all) were processed as 0. Only the items with a CVI of 0.78 or higher were selected [43], and any opinions about additional items and revisions were considered.

The CVI of the 32 preliminary items ranged from 0.80 to 1.00 (19 items with CVI of 1.00, 12 with CVI of 0.90, and 1 with CVI of 0.80), and the CVI was above the cutoff of 0.78 for all items [43]. Items that were suggested to be divided into two items were revised accordingly. After revising terms and phrases and adding and subdividing items, a total of 41 items were generated.

#### 2.2.3. Preliminary Study

To enhance the fit of the instrument by reflecting various opinions considering the facility size, a pilot study was conducted with each 10 family members at a facility with up to 29 beds, 30–99 beds, and 100 or more beds, respectively.

A total of 12 men and 18 women participated in the pilot study conducted from March to April 2018. The mean age was 53.1 years, and the mean duration of caregiving prior to institutionalization was 51.0 months, 21 were college graduates or higher. Older adults’ mean length of stay in the NH was 48.4 months, and the mean number of participants’ monthly visits to the NH was 3.7. At each visit, 18 participants stayed for 1 h or longer.

We asked participants about items that were difficult to understand or answer and about the time needed to respond. There were no problems with comprehensibility, time needed for response, item arrangement, and appropriateness of item length in the pilot study; thus, the main survey was conducted with 41 items.

### 2.3. Data Collection and Ethical Considerations

This study was approved by the institutional review board (IRB No. HYI-17-085-1) at the researcher’s affiliated university. Data were collected from July to October 2018 at 14 NHs (four NHs with 29 beds or less, six NHs with 30–99 beds, four NHs with 100 or more beds) in Seoul, Gyeonggi, Gangwon, Gyeongbuk, and Chungnam, Korea.

In order to reduce measurement errors, a preliminary study was conducted to confirm item comprehension, time needed for response, item arrangement, and appropriateness of item length. Two research assistants were then trained to assist with both the distribution and collection of the questionnaires in the participating nursing homes. In addition, since some items of the tool included a description of the facility staff’s capabilities, self-filled surveys were conducted anonymously in a quiet, independent space so that the response was not affected by the staff.

Prior to data collection, the participants were adequately informed about the purpose and procedures of the study, study participants’ rights, voluntary participation, and confidentiality, and data were collected from those who voluntarily signed the written consent form. In addition, the survey was conducted 2 weeks after initial survey for test–retest.

### 2.4. Instrument

Criterion validity was tested using the Family Perceptions of Care Tool (FPCT) originally developed by Mass and Buckwalter [44] and translated into Korean by Park [45]. It was used as evidence that families with a good cooperative relationship with the facility staff have high satisfaction with care provided by the facility [18,46,47,48]. This tool measures family’s perceptions of care in four aspects (staff consideration, management effectiveness, physical care, activities). The Cronbach’s α was 0.94 in Park’s [45] study and 0.88 in this study.

### 2.5. Statistical Analysis

Data analyses were performed using the SPSS/WIN program version 25.0 (IBM Corp, Armonk, NY, USA) and the AMOS/WIN program version 25.0.

The 330 participants recruited for the man survey were randomized into an EFA or a CFA group (165 in each) using the random case sampling feature of SPSS, according to the study by Hinkin [34], who suggested that different sets of participants should be used for EFA and CFA. Participant’s general characteristic were analyzed using frequency, percentage, mean, and standard deviation. The homogeneity between the EFA and CFA groups was analyzed with t-tests and *χ*^2^ tests. For item analysis, the mean, kurtosis, and skewness of each item were examined, and items with an item-total correlation coefficient above 0.30 were selected [49]. The Kaiser-Meyer-Olkin (KMO) test and Bartlett’s test of sphericity were performed to determine whether the data were appropriate for EFA. In EFA, factors were extracted with principal component analysis (PCA) with Varimax rotation. Factors with an eigenvalue greater than 1.00 were extracted, and items with a commonality greater than 0.40 and factor loading (FL) greater than 0.50 were selected [50].

In the CFA, the criteria for model fitness were as follows: *χ*^2^ (*p*) (*p* > 0.05), normed *χ*² (CMIN/df) ≤ 3, goodness-of-fit index (GFI), and adjusted GFI (AGFI) ≥ 0.80, comparative fit index (CFI) and normed fit index (NFI) ≥ 0.90, root mean square residual (RMR) ≤ 0.05, and root mean square error of approximation (RMSEA) ≤ 0.10 [50]. The criteria for convergent validity were as follows: FL ≥ 0.50; composite reliability (CR) ≥ ± 1.97 (*p* < 0.05); average variance extracted (AVE) ≥ 0.50; and composite construct reliability (CCR) ≥ 0.70. The discriminant validity was tested using AVE and square of correlation coefficient between variables (Φ2). The criterion for discriminant validity was AVE > Φ2 [51].

As for criterion validity, concurrent validity was assessed using Pearson’s correlation analysis with families’ satisfaction with the care provided at NHs, as families with a good cooperative relationship with NH staff were found to have high satisfaction with the care provided [18,46,47,48].

Reliability was verified with item-total correlation (ITC) and internal consistency (Cronbach’s α). The stability of the instrument was analyzed by the test–retest reliability was assessed using intra-class correlation coefficient (ICC).

## 3. Results

### 3.1. Validity and Reliability Testing

#### 3.1.1. Participant Characteristics

The mean age was 53.67 (SD ± 11.04) years, and 62.1% were women. The majority of the participants (80.3%) considered their economic status to be middle class, 45.8% perceived themselves to be in moderate health, and 70.9% reported high stress. Forty percent of the participants were in a 30–99-bed facility, and 60.0% were adult children of the older adult residents. The mean duration of caregiving prior to institutionalization was 53.52 (SD ± 89.85) months. There were no significant differences in the general characteristics between the EFA and CFA groups (Table 1).

#### 3.1.2. Item Analysis

The mean score for each item ranged from 2.90 to 3.66, with SD of 0.49–0.83. After deleting 14 items (#2, #4, #7, #14, #15, #17, #23, #25, #30, #31, #33, #38, #40, #41) with an item-total correlation coefficient below 0.30 [49], we decided to perform factor analysis on the 27 items.

#### 3.1.3. Construct Validity

The construct validity of the scale was evaluated with EFA and CFA and assessing convergent and discriminant validity.

##### EFA

Prior to the EFA, we performed the KMO test and Bartlett’s sphericity test. The KMO value was 0.94, indicating adequacy for factor analysis [45], and Bartlett’s sphericity value was also statistically significant (*χ*^2^ = 2252.85, *p* < 0.001).

A PCA with Varimax rotation was performed to extract the factors. One item with a commonality of below 0.40 (#8), two items with FL of below 0.50 (#9, #13), two items found to the presence of cross-loading (#1, #24) [50], and two items found to have heterogeneous properties relative to other items in terms of the construct (#5, #11) were deleted.

After deleting these items, EFA was performed with the 20 remaining items. The FL was above 0.50 for all items; thus, no additional items were removed. Three factors had an eigenvalue of 1.00 or higher, and there were three significant factors per elbow point on the Scree plot. Furthermore, the explained cumulative variance of these factors was 65.8%, based on which the number of factors was set to three. The first factor explained 30.4%, the second 22.1%, and the third 13.3% (Table 2).

##### CFA

**Figure 2:**
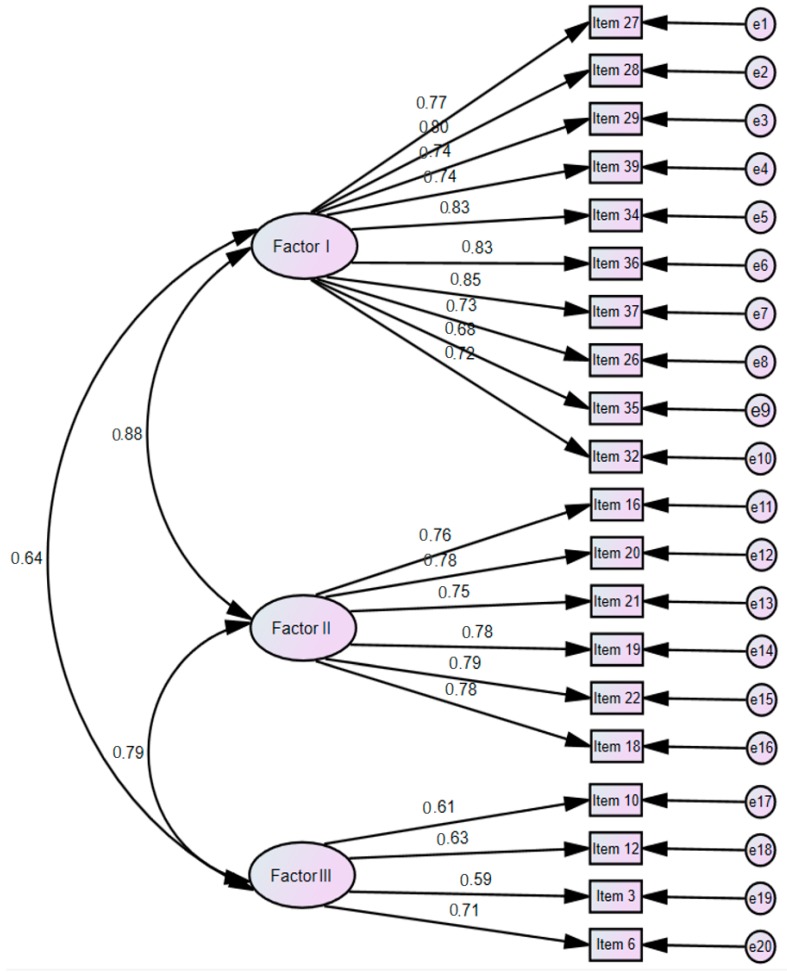
Confirmatory factor analysis of SPIC-F (n = 165) notes. X² (*p*) = 321.72 (*p* < 0.001), df = 167, CMIN/df = 1.93, GFI = 0.84, AGFI = 0.80, CFI = 0.93, NFI = 0.86, RMR = 0.02, RMSEA = 0.08. CMIN/DF = chi-square minimum/degree of freedom; GFI = goodness of fit index; AGFI = adjusted goodness of fit index; CFI = comparative fit index; RMR = root mean square residual; RMSEA = root mean square error of approximation.

CFA was performed to test the construct validity by verifying the number of latent variables and inter-item relationships for the 20 items under the three factors identified through EFA (Figure 2).

We checked whether the items had FL of 0.50 or higher [52] and CR (which determines the significance of FL) of ± 1.965 or higher (*p* < 0.05) [51], and all items satisfied these criteria.

With the exception of *χ*² (*p*), all fitness indices for the final 20 items satisfied the recommended cutoff requirements: *χ*² = 321.72 (*p* < 0.001), normed *χ*² (CMIN/df) = 1.93, GFI = 0.84, AGFI = 0.80, CFI = 0.93, RMR = 0.02, RMSEA = 0.08, and NFI = 0.86.

Subsequently, we assessed convergent validity, which represents the consistency of the items that measure the latent variable. The cutoff for standardized FL (≥0.50) was satisfied with a range of 0.59–0.85, and the cutoff for CR (>1.965) was also satisfied with a range of 5.87–12.50. The cutoff for AVE (>0.50) was met with a range of 0.68–0.81, and so was the cutoff for CCR (>0.70) with a range of 0.89–0.97; therefore, the convergent validity of the scale was established. Finally, to determine the independence of the factors, discriminant validity was tested with AVE > Φ2. The AVE values for Factor 1 (0.79) and Factor 2 (0.81) were greater than the square of the highest correlational coefficient between the two factors (0.78); thus, the scale’s discriminant validity was established.

#### 3.1.4. Criterion Validity

For criterion-related validity, concurrent validity using Pearson’s correlation coefficient test between the SPIC-F and the FPCT. The correlation coefficient was 0.64 (*p* < 0.001), indicating a strong positive correlation between the two instruments, thereby verifying the criterion validity of the SPIC-F (Table 3).

#### 3.1.5. Reliability

The ITC and Cronbach’s α were assessed to verify the internal consistency of the instrument (Table 2). The ITC were all > 0.40 with a range of 0.44–0.79 [49], and Cronbach’s α was 0.95 for the entire 20 items; 0.93 for Factor 1, 0.91 for Factor 2, and 0.74 for Factor 3, all of which were above the cutoff of 0.70 [36]. The ICC was 0.83 (95% CI [0.62, 0.92]) indicating good to excellent retest stability.

### 3.2. Finalization of Scale

Finally, the SPIC-F developed in this study contained 20 items with three factors: professional caring and support (10 items), cooperative relationship and information sharing (6 items), and participation in care (4 items). The average time to complete the survey was about 5–10 min. Items were rated on a 4-point Likert scale and the total score range was 20–80, with higher scores indicating higher levels of partnership between families of NH residents and staff. The SPIC-F scale is attached in Appendix A.

## 4. Discussion

In this study, we identified the components of partnership between families of older adult NH residents and staff and developed an instrument to measure this partnership according to the tool development guideline by DeVellis [36]. The results confirmed that the developed instrument had acceptable validity and reliability.

In our review of the literature, we found that the construct of partnership has been mostly researched in relation to the parents of children and caregivers in acute hospitals [22,23,24,25,28,29], and that the studies on families of older individuals involved those living at home and in care facilities [11,19]. Thus, FGIs were conducted with families of older adult NH residents to obtain a clear understanding of partnership and identify factors that reflect the characteristics of NHs. According to the main properties of partnership identified through FGIs, families of older adult NH residents perceived that a partnership with the staff should involve a mutually respectful and equal relationship, sharing information about care, cooperating to make decisions related to care, and being respected in their decisions. Furthermore, families perceived the provision of consistent care without frequent changes of caregiver as important, wished to know the type of care provided or not provided at the NH for better decision-making, and thought that they should fully cooperate with the care provided at the NH. This highlights the need for more communication between the staff and families of older adult residents in order to build an effective partnership [53].

The features of the SPIC-F are as follows. First, we randomized the participants to the EFA or CFA group because the differences in item variance may disappear in correlation analysis because all items are standardized to common variance if EFA and CFA are performed on the same set of subjects when assessing an instrument’s validity [34]. Thus, we attempted to establish a more appropriate validation process by using two different samples. Second, a variety of analyses were performed to test of validity and reliability of the instrument. EFA and CFA were performed to test the construct validity. Although the p-value for *χ*^2^ was below the cutoff of 0.05 in the CFA for testing the fit of the model, we nevertheless determined that the model fit satisfied the criteria because *χ*^2^ (*p*) may be inappropriate even in models with a good fit due to the complexity of the model or influence of the method of estimation; thus, it should not be trusted unconditionally [51]. We established convergent and discriminant validity, and offered strong evidence for the use of the instrument by confirming its criterion validity, internal consistency, and stability. Third, as the tool consists of 20 items, it is convenient and easy to use. Furthermore, the preliminary survey has made the items easier to comprehend, which can minimize non-response rates. However, because some items of the tool contain contents about the competence of the facility’s staff, it is suggested that they be measured by the facility manager or by a third party rather than by the staff who provide direct care to prevent the Hawthorne effect.

The implications for the application of the SPIC-F are as follows. First, the three factors of SPIC-F consist of items reflecting independent and shared roles of families and staff. Considering that partnership involves collaborating to reach a shared goal while acknowledging each other’s expertise and agreeing on roles and shared responsibilities [54], and that the SPIC-F encompasses contents about independent and shared roles of families and staff, it could be utilized to develop education and intervention programs to build an efficient partnership. In other words, SPIC-F measures each sub-factor or item to find out the lack of family participation, staffs’ professional caring and support, and mutual cooperation, communication, and information sharing among family and staff. On the basis of this, it will be possible to develop a program that can focus on the aspects that are lacking and improve them.

Particularly, through care involvement programs for families of older adult residents, the staff can identify older adults’ unmet medical, emotional, and social needs, which in turn could promote older adults’ participation in activities [18]. Moreover, the quality of life of older adults in NHs would be enhanced and the conflicts between family member and staff would be reduced by encouraging communication between families and staff, helping families to fulfill their roles in caregiving, and having them provide personal care while visiting the NH [55]. Second, although the SPIC-F measures the level of partnership from the families’ perspective, its results could also be utilized when educating facility staff. That is, a low partnership score for the roles of staff could be reflected in the education of staff. Through education, staff would be able to assist older adult residents with their activities of daily living, such as dressing, bathing, or eating, while taking into consideration their needs and preferences [55], promoting person and family-centered care of older adults in NHs [56]. Finally, partnership formation can be used as an indicator of quality care for facility care and can be used as a basis for evaluating and preparing improvement plans. This could also contribute to streamlining health insurance expenditures by further reducing the use of health care by improving the quality of care for the facility.

A limitation of this study is that we used convenience sampling to recruit our participants; thus, this instrument should be validated with subjects from various regions. Furthermore, the instrument was developed in Korea and thus includes Korean cultural features; therefore, additional studies are needed to examine whether it can be utilized in other cultures.

## 5. Conclusions

We developed and validated an instrument to measure partnership between staff and families of older adult NH residents as perceived by families. The SPIC-F consists of 20 items in three categories: professional caring and support (10 items), cooperative relationship and information sharing (6 items), and participation in care (4 items). Each item is rated on a 4-point Likert scale, and the total score range is 20–80, with a higher score indicating a higher level of partnership between staff and families. This instrument was developed by identifying the components of partnership between staff and families and high validity and reliability were established. This instrument is useful for assessing partnership and can be used as a basis for the development and implementation of interventions to effective partnership formation between staff and families of older adult NH residents.

## Figures and Tables

**Table 1:** General characteristics of participants. *N* = 330.

Variables	Category	Total	Group A for EFA (n = 165)	Group B for CFA (n = 165)	t or χ^2^ (*p*)
		n (%) or Mean ± SD			
Age (year)		53.67 ± 11.04	54.85 ± 10.44	52.50 ± 11.51	1.94 (0.053)
Gender	Female	205 (62.1)	105 (63.6)	100 (60.6)	0.32 (0.570)
	Male	125 (37.9)	60 (36.4)	65 (39.4)	
Education	≤Middle school	19 (5.7)	8 (4.8)	11 (6.7)	0.51 (0.773)
	High school	90 (27.3)	45 (27.3)	45 (27.3)	
	≥College	221 (67.0)	112 (67.9)	109 (66.0)	
Perceived economic status	Good	28 (8.5)	16 (9.7)	12 (7.3)	3.40 (0.183)
	Moderate	265 (80.3)	126 (76.4)	139 (84.2)	
	Poor	37 (11.2)	23 (13.9)	14 (8.5)	
Perceived health status	Good	43 (13.0)	23 (13.9)	20 (12.1)	0.40 (0.817)
	Moderate	151 (45.8)	73 (44.3)	78 (47.3)	
	Poor	136 (41.2)	69 (41.8)	67 (40.6)	
Perceived stress status	Low	96 (29.1)	46 (27.9)	50 (30.3)	0.24 (0.628)
	High	234 (70.9)	119 (72.1)	115 (69.7)	
Size of facilities	≤29 beds	28 (8.5)	11 (6.7)	17 (10.3)	3.55 (0.170)
	30–99 beds	132 (40.0)	61 (37.0)	71 (43.0)	
	≥100 beds	170 (51.5)	93 (56.3)	77 (46.7)	
Relationship to older adult resident	Spouse	16 (4.8)	8 (4.8)	8 (4.8)	5.24 (0.388)
	Adult child	198 (60.0)	105 (63.6)	93 (56.4)	
	Daughter-in-law	59 (17.9)	30 (18.2)	29 (17.6)	
	Son-in-law	23 (7.0)	11 (6.7)	12 (7.3)	
	Others	34 (10.3)	11 (6.7)	23 (13.9)	
Duration of caring at home (month)		53.52 ± 89.85	51.47 ± 86.13	55.56 ± 93.65	−0.41 (0.680)

EFA = Exploratory factor analysis; CFA = Confirmatory factor analysis; *p* = Level signification.

**Table 2:** Item analysis and factor analysis of Scale for Partnership In Care—for Family (SPIC-F).

Factor/Item Contents	Mean ± SD	Factor Loadings			Commonality	Explained Variance (%)	ITC	Cron- Bach’s α If Item Deleted	Cron- Bach’s α	ICC (95%CI) (n = 27)	AVE	CCR
		1	2	3								
**Factor 1**						**30.4**			**0.93**	**0.80** **(0.57–0.91)**	**0.79**	**0.97**
27. Staff encourage the family to visit the facility.	3.08 ± 0.75	**0.79**	0.18	0.16	0.68		0.70	0.95				
28. Staff positively support family involvement in providing care (e.g., conversation, taking a walk, meal assistance, etc.).	3.26 ± 0.58	**0.78**	0.20	0.19	0.69		0.74	0.95				
29. Staff welcome the family when they visit the facility.	3.28 ± 0.69	**0.75**	0.26	0.17	0.66		0.70	0.95				
39. Staff inform the family about the regulations and the policies of the facility before he or she is admitted.	3.31 ± 0.59	**0.74**	0.18	0.22	0.64		0.70	0.95				
34. Staff respect and support the families’ decision-making on the older adults residing in the facility.	3.22 ± 0.60	**0.71**	0.40	0.17	0.69		0.77	0.94				
36. Staff provide appropriate care on the condition of the older adults residing in the facility.	3.23 ± 0.58	**0.71**	0.46	0.17	0.74		0.79	0.94				
37. Staff provide care while maintaining the dignity of the older adults residing in the facility.	3.20 ± 0.61	**0.70**	0.35	0.26	0.68		0.77	0.95				
26. Staff inform the family about the condition or changes in the condition of the older adults residing in the facility.	3.35 ± 0.59	**0.67**	0.40	0.19	0.65		0.74	0.95				
35. Staff are sensitive to changes in the state of the older adults residing in the facility.	3.10 ± 0.69	**0.67**	0.34	0.00	0.56		0.63	0.95				
32. Staff involve families when planning care for the older adults residing in the facility.	2.95 ± 0.68	**0.63**	0.31	0.06	0.50		0.65	0.95				
**Factor 2**						**22.1**			**0.91**	**0.71** **(0.36–0.87)**	**0.81**	**0.96**
16. Staff and I communicate smoothly regarding caring for the older adult.	3.28 ± 0.60	0.32	**0.77**	0.22	0.76		0.74	0.95				
20. Staff and I discuss the range of roles that each other should take in caring for the older adult.	3.14 ± 0.64	0.33	**0.77**	0.22	0.75		0.73	0.95				
21. Staff and I respect each other’s knowledge and experience with regard to caring for the older adults residing in the facility.	3.30 ± 0.57	0.36	**0.70**	0.30	0.70		0.71	0.95				
19. Staff and I understand and sympathize with each other’s difficulties in caring for the older adults residing in the facility.	3.29 ± 0.57	0.49	**0.69**	0.15	0.73		0.75	0.95				
22. Staff and I find solutions together when problems occur regarding the older adults residing in the facility.	3.32 ± 0.60	0.34	**0.68**	0.26	0.65		0.72	0.95				
18. Staff and I share a common goal in caring for the older adults residing in the facility.	3.30 ± 0.63	0.39	**0.67**	0.32	0.71		0.75	0.95				
**Factor 3**						**13.3**			**0.74**	**0.85** **(0.67–0.93)**	**0.68**	**0.89**
10. I am involved in the care of the older adult residing in the facility.	3.24 ± 0.61	0.10	0.09	**0.81**	0.67		0.44	0.95				
12. I pay enough attention to the older adult residing in the facility.	3.27 ± 0.51	0.21	0.15	**0.77**	0.66		0.48	0.95				
3. I provide staff with information on the characteristics of the older adult before he or she is admitted.	3.48 ± 0.57	0.13	0.39	**0.64**	0.57		0.50	0.95				
6. I actively participate when the staff ask for cooperation regarding the older adult residing in the facility.	3.52 ± 0.57	0.19	0.36	**0.56**	0.48		0.51	0.95				
Total							65.8		0.95	0.83 (0.62–0.92)		
KMO = 0.94, Bartlett’s test: *χ*_2_ = 2252.85 (*p* < 0.001)												

ITC = Item-Total Correlation; ICC = Intra-class Correlation Coefficient; AVE = Average Variation Extracted; CCR = Composite Construct Reliability.

**Table 3:** Correlation between SPIC-F and Family Perceptions of Care Tool (FPCT). *N* = 330.

Measurement	SPIC-F	Factor 1	Factor 2	Factor 3
	r (*p*)	r (*p*)	r (*p*)	r (*p*)
FPCT	0.64 (<0.001)	0.68 (<0.001)	0.55 (<0.001)	0.28 (<0.001)

SPIC-F = scale for partnership in care—for family; r = Pearson’s correlation coefficient; *p* = level signification.

## Appendix A. SPIC-F Scale

Subject: family member of older adult nursing home (NH) resident

**Table A1:** The following questions are about partnerships with families and staff. For the following questions, please circle the number that best corresponds to your views.

Item	Strongly Disagree	Disagree	Agree	Strongly Agree
**Professional caring and support**				
1. Staff encourage the family to visit the facility.	①	②	③	④
2. Staff positively support family involvement in providing care (e.g., conversation, taking a walk, meal assistance, etc.).	①	②	③	④
3. Staff welcome the family when they visit the facility.	①	②	③	④
4. Staff inform the family about the regulations and the policies of the facility before he or she is admitted.	①	②	③	④
5. Staff respect and support the families’ decision-making on the older adults residing in the facility.	①	②	③	④
6. Staff provide appropriate care on the condition of the older adults residing in the facility.	①	②	③	④
7. Staff provide care while maintaining the dignity of the older adults residing in the facility.	①	②	③	④
8. Staff inform the family about the condition or changes in the condition of the older adults residing in the facility.	①	②	③	④
9. Staff are sensitive to changes in the state of the older adults residing in the facility.	①	②	③	④
10. Staff involve families when planning care for the older adults residing in the facility.	①	②	③	④
**Cooperative relationship and information sharing**				
11. Staff and I communicate smoothly regarding caring for the older adult.	①	②	③	④
12. Staff and I discuss the range of roles that each other should take in caring for the older adult.	①	②	③	④
13. Staff and I respect each other’s knowledge and experience with regard to caring for the older adults residing in the facility.	①	②	③	④
14. Staff and I understand and sympathize with each other’s difficulties in caring for the older adults residing in the facility.	①	②	③	④
15. Staff and I find solutions together when problems occur regarding the older adults residing in the facility.	①	②	③	④
16. Staff and I share a common goal in caring for the older adults residing in the facility.	①	②	③	④
**Participation in care**				
17. I am involved in the care of the older adult residing in the facility.	①	②	③	④
18. I pay enough attention to the older adult residing in the facility.	①	②	③	④
19. I provide staff with information on the characteristics of the older adult before he or she is admitted.	①	②	③	④
20. I actively participate when the staff ask for cooperation regarding the older adult residing in the facility.	①	②	③	④
